# The Autoimmune/Inflammatory Syndrome Induced by Adjuvants (ASIA), Associated with Renal Compromise and Cutaneous Calcinosis: A Case Report and Literature Review

**DOI:** 10.1155/2024/7524714

**Published:** 2024-05-14

**Authors:** Cristian Betancur Henao, Juan Guillermo Rifaldo, Rafael Vicente-Pérez, Maria Cristina Martinez-Avila, Rodrigo Daza-Arnedo, Jorge Rico-Fontalvo

**Affiliations:** ^1^Internal Medicine Department, Universidad Cooperativa de Colombia, Medellín, Colombia; ^2^Division of Nephrology, San Juan de Dios Hospital, Santa Fe de Antioquia, Antioquia, Colombia; ^3^Colombian Association of Nephrology and Hypertension, Bogotá, Colombia; ^4^Epidemiology and Public Health, Polytechnic University of Nicaragua, Managua, Nicaragua; ^5^Internal Medicine Department, Universidad del Bosque, Bogotá, Colombia

## Abstract

The autoimmune/inflammatory syndrome induced by adjuvants (ASIA) was first introduced in 2011 to provide a more precise syndromic characterization of clinical manifestations observed in patients exposed to adjuvant substances such as biopolymers and silicone, among others. The clinical spectrum of this entity is variable, ranging from local involvement to potentially fatal immune-mediated systemic involvement. The interest in ASIA has grown in recent years, reinforcing diagnostic criteria and deepening the understanding of its pathophysiological behavior. This case report highlights a distinct range of clinical symptoms, such as general symptoms, advanced-stage chronic kidney disease, persistent hypercalcemia with suppressed parathyroid hormone (PTH), bilateral nephrocalcinosis, cutaneous calcinosis, and the presence of positive autoantibodies, emphasizing the significance of understanding this condition.

## 1. Introduction

The term “silicone” is used generically to refer to a large family of synthetic polymers containing inorganic materials formed through polymerization and cross-linking of their molecules, chemically known as synthetic organosiloxanes [1]. Silicone is initially marketed as an “ideal” aesthetic adjuvant, deemed harmless, immunologically inactive, long-lasting, affordable, and noncarcinogenic [1].

Since its widespread use for aesthetic purposes in the 1940s in Japan, numerous cases have described a range of adverse effects, including autoimmune, granulomatous inflammatory reactions, rheumatologic conditions, and even hematological-oncological issues, collectively termed Siliconosis [2, 3]. The manifestations of Siliconosis can be early, involving local reactions, or late, characterized by autoimmune and inflammatory features. When ASIA was first described in 2011, it encompassed various forms of presentation, including Siliconosis, postvaccination phenomena, macrophagic myofasciitis syndrome, Gulf War syndrome, and sick building syndrome [4]. Following further reviews, they have been grouped into more prevalent forms associated with the underlying pathophysiological mechanism [5]. These include Siliconosis, Sarcoidosis, Sjogren's syndrome, undifferentiated connective tissue disease, and other immune-mediated adverse events [6–9]. ASIA may predispose to the development of these diseases [5].

Currently, there are few reported cases, and even fewer with the clinical conditions presented by our patient. We will discuss the findings in light of the current available evidence.

## 2. Case Description

We present the case of a 44-year-old female with no relevant medical history, admitted due to pelvic pain associated with elevated creatinine and azotemia. The patient had undergone legally liquid silicone biopolymer injections in the gluteal region approximately 20 years ago for aesthetic purposes. Years later, she developed nonspecific symptoms, including fatigue, general discomfort, intermittent myalgias, sporadic insomnia, and chronic pelvic pain. These symptoms partially responded to conventional medical management (NSAIDs). Notably, there were no records of nephropathy before this event.

On admission, the patient was stable, and the physical examination revealed pelvic tenderness with firm, whitish/yellowish papules, plaques, and palpable multiple nodular lesions in the hypogastric, pelvic, gluteal, inguinal, and sacral regions, without local infectious changes. Some nodules restrict joint mobility and limit movement due to stiffening of the skin. Laboratory tests (Table 1) revealed elevated creatinine, hypercalcemia, and urinalysis indicating pyuria and proteinuria.

**Figure 1:**
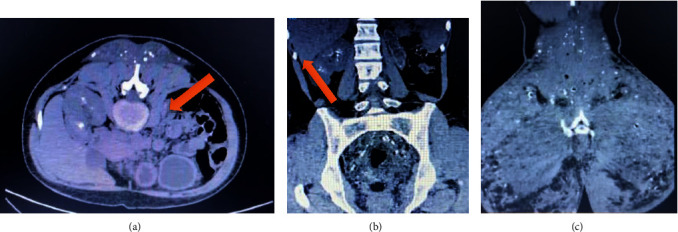
(a) The arrow points to left renal atrophy. (b) The arrow indicates marked nephrocalcinosis in the right kidney. (c) Extensive involvement of soft tissue by cutaneous calcinosis associated with biopolymers.

Initial nephropathy management included optimal fluid therapy, discontinuation of nephrotoxic agents (NSAIDs), and a urotomography (Figures 1(a)–1(c)), which described severe thickening of soft tissues in the bilateral lumbar and gluteal regions with calcifications, pelvic retroperitoneal calcifications, and atrophic left kidney with calcifications. The right kidney showed normal size with intraparenchymal and calyceal calcifications consistent with nephrocalcinosis, without masses or dilation of collecting cavities. With no clear diagnosis, further studies were conducted. A renal artery Doppler confirmed bilateral nephrocalcinosis, a normal right renal artery, and changes in the left kidney secondary to unspecified hypoplasia without thrombosis. Autoimmune profile revealed positive antinuclear antibodies (ANA) with a speckled pattern 1 : 160, negative anti-neutrophil cytoplasmic antibodies (ANCAs), and normal complement levels. Infectious profile (HIV, HBV, HCV) was negative, and additional metabolic profile showed suppressed PTH, elevated 25-hydroxyvitamin D, and normal 1,25-dihydroxyvitamin D. Plasma cell involvement was ruled out through serum immunoelectrophoresis, and long bone X-rays showed no lesions (Table 2).

Given the persistent hypercalcemia, suppressed PTH, and bilateral nephrocalcinosis, the patient was diagnosed with chronic kidney disease and cutaneous calcinosis as sequelae of ASIA. It was decided to initiate treatment with prednisone at 0.5 mg/kg/day, showing a slight decrease in calcium levels but no significant changes in creatinine or azotemia. During the hospital stay, her clinical course was favorable, so she was discharged for further outpatient follow-up.

## 3. Discussion

Since its recognition as a pathological entity, over 4,000 cases of ASIA with similar characteristics have been documented, with varying temporalities, severity, and exposure to different adjuvants [9, 10]. Clinically, although ASIA shares classic manifestations with rheumatological entities such as fibromyalgia, chronic fatigue syndrome, and autoimmune diseases like lupus and Sjögren's syndrome [11–14], our patient did not meet the diagnostic criteria for these conditions. The chronological association of symptoms with the silicone injection event prompted extensive studies to explain the myriad manifestations.

With the findings described and considering the timeline of our patient's history, where the onset of symptoms was clearly linked to the application of biopolymers, studies were initiated to explain the myriad manifestations. Solid tumors were ruled out through questioning, absence of symptoms, and diagnostic imaging. Plasma cell malignancy was considered due to CRAB signs, with normal X-rays and serum and urine immunofixations showing no monoclonal spikes. There were no other relevant findings in blood biochemistry suggesting medullary involvement, and there were no adenopathies. Chronic infections such as HIV, HBV, and HCV, as well as thyroid and adrenal disorders, were ruled out.

Granulomatous infiltration due to silicone injections is not rare; therefore, granulomatous diseases were considered, ruling out tuberculosis due to its high prevalence in our region with a normal chest X-ray and negative PPD. Another diagnostic possibility was sarcoidosis, which has been described as an infrequent manifestation within the spectrum of ASIA syndrome, despite the absence of pulmonary, lymph node, or cutaneous involvement, which are more common for this condition. Unfortunately, a histopathological study, necessary for its diagnosis, could not be performed due to the high risk of complications in her context.

Additionally, systemic autoimmune disorders and ANCA-positive vasculitis were ruled out by the negativity of antibodies for these entities, except for ANA, which, while positive, did not meet the ACR/EULAR criteria for systemic lupus erythematosus, and the patient did not have evidence suggesting active connective tissue disease. Having ruled out all previously mentioned conditions, the classic criteria for ASIA syndrome proposed by Shoenfeld and Agmon-Levin were applied, and it was found that 2 major criteria and one minor criterion were met, leading to the diagnosis of ASIA syndrome [4, 15, 16].

Like most autoimmune diseases, patients with ASIA syndrome appear to have a genetic susceptibility (presence of certain genetic haplotypes, including HLA-DR5 and HLA DQ2) that makes them more prone to developing symptoms in response to a specific trigger [17], in this case, exposure to silicon-containing compounds. After silicone implantation, a capsule forms around the implant as part of an inflammatory response to a foreign body, triggering a local reaction. However, in genetically susceptible individuals, this inflammatory response may be uncontrolled, leading to the production of autoantibodies and occasionally an autoimmune disease with systemic repercussions [10, 18].

The exact underlying immunological mechanism is not known with certainty. It is believed that silicone and similar compounds act as adjuvants rather than direct antigens, meaning molecules that, on their own, do not trigger an inflammatory response through direct stimulation of T lymphocytes but rather enhance an already initiated reaction. Some proposed mechanisms include enhancing the local reaction at the injection site by mimicking DAMPS (damage-associated molecular patterns), constant stimulation leading to increased exposure of antigen-presenting cells, promoting the translocation of antigens to lymph nodes, and possibly their hydrophobicity, favoring the accumulation of plasma proteins on the surface that subsequently activate the immune system [6].

There are multiple cases in the literature with similar characteristics; however, to date, only one reported case shares the clinical spectrum of chronic kidney disease, calcinosis cutis, and persistent hypercalcemia [10].

The exact mechanism of renal damage in patients exposed to silicones is still unclear. Although it has been correlated with a granulomatous disease associated with siliconomas or paraffinomas that explains inflammation confined to tubulointerstitial behavior, interstitial and membranous nephritis has been described without evidence of granuloma [19]. Despite significant advances in the pathophysiological understanding of ASIA syndrome, more reports are unfortunately still needed to clarify the specific mechanism explaining renal impairment in these patients. In the mentioned case, renal biopsy was not possible due to significant renal atrophy and a valuable single kidney with a high risk of complications due to extensive nephrocalcinosis.

Calcinosis cutis, a phenomenon frequently seen in patients with chronic kidney disease, cancer, and autoimmune conditions such as CREST syndrome (calcinosis cutis, Raynaud's phenomenon, esophageal dysmotility, sclerodactyly, and telangiectasia), is explained by the exposure of denatured proteins in damaged tissue, allowing the interaction between calcium and phosphate to form calcium phosphate crystals that perpetuate tissue damage and lead to the formation of giant accumulations [20]. These accumulations are often the focus of intense granulomatous fibrosis that limits the tissue repair process [20]. In our patient, the cutaneous involvement associated with calcinosis cutis was very striking because it extended beyond the area of silicone application in the gluteal region to the lumbar region and was even evident in the distal pubic, thigh, and soft tissues in the dorsal region.

The silicone injection event likely triggered a cascade of immune-mediated reactions, leading to systemic inflammation and tissue damage. The association between silicone injection, hypercalcemia, and granulomatous disease has been widely described since the 1980s [21] and has been explained by increased activity in the 1a-hydroxylase enzyme of giant cells and macrophages leading to increased production of 1,25-dihydroxycholecalciferol. Elevated levels of parathyroid hormone-related protein (PTHrP) have also been found at the expense of macrophages and giant cells in patients with hypercalcemia and granulomatous disease due to silicones. These two hypotheses are supported by the excellent response of hypercalcemia to glucocorticoid therapy, which has been shown to be effective in reducing levels of 1,25-dihydroxycholecalciferol and PTHrP [22].

Importantly, given the paucity of reported cases, there is a lack of standardized treatment protocols for ASIA, with most management strategies being empiric and symptom based. Corticosteroids have been shown to control the overproduction of 1,25-dihydroxycholecalciferol as a potent inhibitor of the 1a-hydroxylase enzyme, in addition to reducing PTHrP levels through cytokine production control in macrophages (mainly IL-6 and IFN-gamma) [4]. On the other hand, hydroxychloroquine and ketoconazole, which have been effective in treating hypercalcemia in patients with sarcoidosis, have shown a lower rate of adverse events than prolonged glucocorticoid therapy and could be an alternative in patients with silicone-induced hypercalcemia [4]. However, further studies are still needed to support this theory.

This case is unique due to the combination of symptoms, including advanced-stage chronic kidney disease, cutaneous calcinosis, and the presence of positive autoantibodies. The relationship between silicone exposure and autoimmune phenomena, as well as the underlying pathophysiological mechanisms, warrants further research. This case emphasizes the importance of considering ASIA in patients with a history of adjuvant exposure presenting with multisystemic manifestations, as early recognition and intervention may prevent progression to irreversible organ damage.

## 4. Conclusion

ASIA is a complex and evolving clinical entity characterized by diverse manifestations related to adjuvant exposure. This case report highlights a unique presentation of ASIA with advanced-stage chronic kidney disease, persistent hypercalcemia, bilateral nephrocalcinosis, and cutaneous calcinosis. The recognition of ASIA and its potential complications is crucial for guiding appropriate diagnostic and therapeutic interventions. Further research is needed to elucidate the underlying pathophysiological mechanisms and establish standardized management protocols for this challenging condition.

## Figures and Tables

**Table 1:** Laboratory test.

Urinalysis	pH: 6 Specific gravity: 1.020 Sediment: RBCs 0, WBCs 15–20/HPF, leukocyte casts 6–8/HPF, abundant bacteria
Blood urea nitrogen	43 mg/dL (5–20) (estimated GFR by CKD-EPI: 17 ml/min/1.73 m^2^)
Creatinine	3.1 mg/dL (0.7–1.3)
Chloride	107.1 mmol/L (95–106)
Sodium	135.5 mmol/L (135–145)
Potassium	4.4 mmol/L (3.5–5.0)
Serum calcium	12.6 mg/dL (8.5–10.0)
Phosphorus	4.5 mmol/L (1.2–1.45)
Hemogram	(i) Hb: 10.4 g/dL (12–15) (ii) Hematocrit: 33% (35–45%) (iii) Mean corpuscular volume (VCM): 87 (80–100) (iv) White blood cells: 15,200 (4,500−10,000) (v) Neutrophils: 9,790 (vi) Lymphocytes: 4,330 (vii) Platelets: 249,000 (150,000–450,000/mm^3^)
Albumin	4.1 g/dL (3.5–5.5)
24-hour urine protein	1.934 mg/day (<150 mg/day)
Glucose	91.6 mg/dL (70–100)
C-reactive protein	<0.5 mg/dL (<0.5)
ALT (alanine aminotransferase)	30 U/L (7–50)
AST (aspartate aminotransferase)	66.7 U/L (5–40)
Bilirubin	Total 0.2 mg/dl, indirect 0.2 mg/dL
ANA (antinuclear antibody)	Positive, speckled pattern, 1 : 160
ANCAs by IFI	Negative
Anti-double-stranded DNA antibodies	Negative
Anti-La antibodies	2.40 (negative)
Anti-Ro antibodies	10.30 (negative)
Anti-SM antibodies	3.50 (negative)
Anti-RNP antibodies	1.10 (negative)
Complement C4	24.5 (consumed)
Vitamin D 25-hydroxy	17.2 ng/mL (20–40)
Vitamin 1,25 dihydroxyvitamin D	12.5 pg/mL (18–78)
PTH (parathyroid hormone)	5.12 pg/mL (15–65)
PTHrP (parathyroid hormone-related protein)	12.4 pmol/L (0.0–2.0)
Cortisol (8 am morning)	267 nmol/L (140–690)
TSH (thyroid stimulating hormone)	2.09 mUI/L (0.3–4.7)
Serum protein immunoelectrophoresis	No detectable monoclonal component
PPD (purified protein derivative)	Negative
HIV	Negative
Hepatitis B and C viruses	Negative

**Table 2:** Image interpretation.

Skull X-ray	No lesions

Long bone X-ray	Preserved bone mineral density. No suggestive images of fractures, lytic, or blastic lesions

Doppler of renal arteries	Bilateral nephrocalcinosis changes observed
	On the right renal side, no turbulence zones are observed, and the wave spectrum is normal
	On the left side, there are widespread late parvus waves. No renal vein thrombosis detected

## Data Availability

The data used to support the findings of this study are included within the article.

## References

[1] Narins R. S., Beer K. (2006). Liquid injectable silicone: a review of its history, immunology, technical considerations, complications, and potential. *Plastic and Reconstructive Surgery*.

[2] Israeli E., Agmon-Levin N., Blank M., Shoenfeld Y. (2009). Adjuvants and autoimmunity. *Lupus*.

[3] Borba V., Malkova A., Basantsova N. (2020). Classical examples of the concept of the ASIA syndrome. *Biomolecules*.

[4] Shoenfeld Y., Agmon-Levin N. (2011). ASIA–autoimmune/inflammatory syndrome induced by adjuvants. *Journal of Autoimmunity*.

[5] Watad A., Sharif K., Shoenfeld Y. (2017). The ASIA syndrome: basic concepts. *MJR*.

[6] McKee A. S., Munks M. W., MacLeod M. K. (2009). Alum induces innate immune responses through macrophage and mast cell sensors, but these sensors are not required for alum to act as an adjuvant for specific immunity. *The Journal of Immunology*.

[7] Ruyer-Thompson M., Guérin A., Borba V. (2021). The downside of beauty: ASIA syndrome associated with local silicone injections: a literature review. *Immunome Research*.

[8] Cojocaru M., Chicos B. (2013). ASIA or Shoenfeld’s syndrome an autoimmune syndrome induced by adjuvants. *Romanian Journal of Internal Medicine*.

[9] Jara L. J., García-Collinot G., Medina G. (2016). Severe manifestations of autoimmune syndrome induced by adjuvants (Shoenfeld’s syndrome). *Immunologic Research*.

[10] Barilaro G., Spaziani Testa C., Cacciani A., Donato G., Dimko M., Mariotti A. (2016). ASIA syndrome, calcinosis cutis and chronic kidney disease following silicone injections. A case-based review. *Immunologic Research*.

[11] Baldwin C. M., Kaplan E. N. (1983). Silicone-induced human adjuvant disease?. *Annals of Plastic Surgery*.

[12] Vasey F. B., Zarabadi S. A., Seleznick M., Ricca L. (2003). Where there’s smoke there’s fire: the silicone breast implant controversy continues to flicker: a new disease that needs to be defined. *Journal of Rheumatology*.

[13] Fryzek J. P., Signorello L. B., Hakelius L. (2001). Self-reported symptoms among women after cosmetic breast implant and breast reduction surgery. *Plastic and Reconstructive Surgery*.

[14] Maijers M. C., de Blok C. J., Niessen F. B. (2013). Women with silicone breast implants and unexplained systemic symptoms: a descriptive cohort study. *The Netherlands Journal of Medicine*.

[15] Camuzard O., Dumas P., Foissac R. (2014). Severe granulomatous reaction associated with hypercalcemia occurring after silicone soft tissue augmentation of the buttocks: a case report. *Aesthetic Plastic Surgery*.

[16] Ortiz-Álvarez J., Lebron-Martin J. A., Rodriguez Fernandez-Freire L., Zulueta-Dorado T., Garcia-Morillo J. S. (2021). Cutaneous and ganglion sarcoidosis induced by polycaprolactone facial filler: a new expression of ASIA syndrome?. *European Journal of Case Reports in Internal Medicine*.

[17] Young V. L., Nemecek J. R., Schwartz B. D., Phelan D. L., Schorr M. W. (1995). HLA typing in women with breast implants. *Plastic and Reconstructive Surgery*.

[18] Cohen Tervaert J. W. (2018). Autoinflammatory/autoimmunity syndrome induced by adjuvants (ASIA; Shoenfeld’s syndrome): a new flame. *Autoimmunity Reviews*.

[19] Agrawal V., Crisi G. M., D’Agati V. D., Freda B. J. (2012). Renal sarcoidosis presenting as acute kidney injury with granulomatous interstitial nephritis and vasculitis. *American Journal of Kidney Diseases*.

[20] Buchowski J. M., Ahn N. U., Ahn U. M., McCarthy E. F., Mehta M. B. (2001). Disproportionately severe calcinosis cutis in an 88-year-old patient with CREST syndrome. *Skeletal Radiology*.

[21] Kozeny G. A., Barbato A. L., Bansal V. K., Vertuno L. L., Hano J. E. (1984). Hypercalcemia associated with silicone-induced granulomas. *New England Journal of Medicine*.

[22] Fuss M., Pepersack T., Gillet C., Karmali R., Corvilain J. (1992). Calcium and vitamin D metabolism in granulomatous diseases. *Clinical Rheumatology*.

